# Cost-Utility Analysis of Lipegfilgrastim Compared to Pegfilgrastim for the Prophylaxis of Chemotherapy-Induced Neutropenia in Patients with Stage II-IV Breast Cancer

**DOI:** 10.3389/fphar.2017.00614

**Published:** 2017-09-13

**Authors:** Esse I. H. Akpo, Irshaad R. Jansen, Edith Maes, Steven Simoens

**Affiliations:** ^1^Market Access Strategy and Health Economics Deloitte (Belgium), Zaventem, Belgium; ^2^Department of Pharmaceutical and Pharmacological Sciences KU Leuven, Leuven, Belgium

**Keywords:** lipegfilgrastim, pegfilgrastim, breast cancer, cost-utility, febrile neutropenia, severe neutropenia

## Abstract

**Background:** Lipegfilgrastim (Lonquex®) has demonstrated to be non-inferior to pegfilgrastim (Neulasta®) in reducing the duration of severe neutropenia (SN) in patients with stage II−IV breast cancer. Compared to pegfilgrastim, lipegfilgrastim also demonstrated statistically significant lower time to ANC recovery in cycles 1–3, lower incidence of SN in cycle 2 and lower depth of absolute neutrophil count (ANC) nadir in cycles 2 and 3. The aim of this study was to quantify the cost utility of lipegfilgrastim compared to pegfilgrastim in stage II−IV breast cancer patients, taking the perspective of the Belgian payer over a lifetime horizon.

**Methods:** Two Markov models were developed to track on- and post-chemotherapy related complications, including SN, febrile neutropenia (FN), chemotherapy dose delay, chemotherapy relative dose intensity of less than 85%, infection, death rates, and quality-adjusted life years (QALYs). Data on costs (2015 value) and effects were obtained from literature, national references, and complemented by a survey of clinical experts using a modified Delphi method. Both deterministic and probabilistic sensitivity analyses were carried out. Outcomes measures included costs, QALYs and life-years (LY).

**Results:** At current equivalent price of €1,169, treatment with lipegfilgrastim was associated with overall costs of €9,845 vs. €10,208 for pegfilgrastim and overall QALYs of 13.977 vs. 13.925 for pegfilgrastim. Life expectancy was increased by 21 days (or 0.058 LY gained). The difference in costs stem from avoided infection, SN and FN cases in the lipegfilgrastim compared to the pegfilgrastim group. Similarly, the difference in QALYs was explained by the difference in the number of patients in the chemotherapy/G-CSF Markov state followed by infection and FN between lipegfilgrastim and pegfilgrastim. The probability of lipegfilgrastim to be cost-effective compared to pegfilgrastim was 68, 79, and 83% at the willingness-to-pay thresholds (WTP) of €10,000, €30,000 and €50,000 per QALY gained, respectively. At a WTP threshold of €30,000 per QALY gained, lipegfilgrastim was cost-effective up to €1,500 across all age bands and cancer stages, compared to the current price.

**Conclusions:** Lipegfilgrastim is a cost-effective use of health care resources in patients with stage II-IV breast cancer.

## Introduction

Chemotherapy-induced neutropenia (CIN), a common side effect of cancer chemotherapy, is a major risk factor for infection-related morbidity and mortality and a significant dose-limiting toxicity (Aapro et al., 2011). The duration and severity of CIN depend on various factors including type of cancer, patient-specific risk factors, individual disease characteristics, and chemotherapy regimen (Crawford et al., 2004). Prolonged and severe CIN may lead to serious complications in the short-term due to the increased risk of febrile neutropenia (FN) and infections. As a result of CIN, the subsequent chemotherapy cycles may be delayed to allow for neutrophil recovery; or the chemotherapy doses may be reduced in an effort to minimize the incidence of CIN in later cycles (Mucenski and Shogan, 2003). Consequently, when CIN occurs, patient's quality of life is impaired and the long-term clinical efficacy and cost-effectiveness of chemotherapy may be compromised (Trillet-Lenoir et al., 1993; Crawford et al., 2004; Lyman, 2009).

To counteract the negative impact of CIN, short and long-acting recombinant granulocyte colony-stimulating factors (G-CSFs) are used to promote the proliferation, differentiation, and maturation of neutrophils, thereby reducing the duration and severity of CIN as well as the incidence of severe neutropenia (SN), FN and infection-related mortality (Crawford et al., 1991; Trillet-Lenoir et al., 1993; Kuderer et al., 2007; Wang et al., 2015). The data suggest that long-acting G-CSFs are more effective compared to short-acting G-CSFs in terms of incidence of FN (Mitchell et al., 2016). They are also less burdensome to administer (once per cycle with long-acting vs. up to 11 injections with short-acting G-CSFs). Therefore, in Belgium, long-acting -G-CSFs are more often used than short-acting G-CSFs. Current market approved long-acting compounds include pegfilgrastim (Neulasta®; Amgen Inc.) and lipegfilgrastim (Lonquex®; Teva Pharmaceuticals Europe B.V.); with the glycopegylated G-CSF lipegfilgrastim presenting distinct pharmacokinetic and pharmacodynamics properties compared to pegfilgrastim (Hoggatt et al., 2015; Guariglia et al., 2016). Lipegfilgrastim has a slower clearance than pegfilgrastim, with a terminal half-life of lipegfilgrastim shown to be 7−10 h longer than that of pegfilgrastim in healthy volunteers (Buchner et al., 2014; Guariglia et al., 2016).

In a randomized, multicenter, active-control phase III trial, Bondarenko et al. demonstrated that lipegfilgrastim was as effective as pegfilgrastim in reducing the duration of SN in patients with breast cancer receiving myelosuppressive chemotherapy in primary prophylaxis (Bondarenko et al., 2013). Wang et al. and Bond et al. further confirmed in meta-analyses that the risk of FN was not significantly different between lipegfilgrastim and pegfilgrastim with an odds ratio (OR) of 0.98 (95% CI: 0.21–4.53) and a relative risk (RR) of 0.34 (95% CI: 0.05–2.14), respectively (Bond et al., 2015; Wang et al., 2015). There were further no differences in hospitalization and antibiotic usage between the two G-CSFs (Gladkov et al., 2016).

However, Bondarenko et al. reported significant lower incidence of SN in cycle 2 of chemotherapy in the lipegfilgrastim group compared to the pegfilgrastim group (8.5% vs. 21.5%; *P* = 0.013). The absolute neutrophil count (ANC) nadir (10^9^/L) after cycles 2-3 was higher in the lipegfilgrastim group compared to pegfilgrastim group (2.6 vs. 2.0 and 2.5 vs. 2.0; *P* = 0.019 and *P* = 0.035, respectively). Similarly, the time to ANC recovery (days) was −1.59 days shorter for the lipegfilgrastim group compared to the pegfilgrastim one (−1.59, −1.66, and −1.34; *P* = 0.003, *P* = 0.008, and *P* = 0.033 for cycles 1, 2, and 3, respectively). In the lipegfilgrastim group, one patient experienced FN compared to three patients in the pegfilgrastim group. Additionally, 31 patients in the lipegfilgrastim group had their chemotherapy delayed in subsequent cycles with no dose omissions or reductions in cycles 2−4. In the pegfilgrastim group, 36 patients received delayed chemotherapy treatment, and eight patients had dose omissions or reductions in cycles 2−4 (Bondarenko et al., 2013).

Cost-effectiveness analysis is increasingly being used to guide resource allocation and maximize health benefits as a result of increasing health expenditures and resulting budgetary constraints. There are very few studies assessing the cost-effectiveness of lipegfilgrastim compared to pegfilgrastim. Taking the Belgian payer perspective, Fust et al. concluded that pegfilgrastim was cost-effective vs. lipegfilgrastim in patients with stage II breast cancer using Wang et al.'s risk estimate for FN of 1.39 (0.54–3.50) for lipegfilgrastim compared to pegfilgrastim (Fust et al., 2015, 2016; Wang et al., 2015). Wang et al.'s risk estimate for FN was derived from a mixed treatment comparison (MTC) using a heterogeneous (i.e., non-breast cancer specific) cancer population and considering different studies designs. Kulikov et al., on the other hand, conducted a comparative pharmacoeconomic analysis of prophylactic use of lipegfilgrastim, pegfilgrastim, filgrastim, and lenograstim in the prevention of FN in Russia's health care setting (Kulikov et al., 2016). Kulikov et al. concluded that lipegfilgrastim was the dominant therapy as it allows to increase the number of patients who responded to prophylaxis of FN while reducing costs as compared to other G-CSFs. Budget impact analysis, considering the costs for G-CSF drugs, costs for the treatment of incurring cases of FN and costs for the management of adverse events showed that the use of lipegfilgrastim for the prevention of FN saves budget costs when compared to all other G-CSF drugs over a 1 year time horizon. The data on drugs efficacy (measured as the proportion of responders to prophylaxis of FN by the end of the first year) was obtained from Bond et al.'s meta-analysis (Bond et al., 2015). It should be noted that Bond et al.'s risk estimate for FN was based on a direct comparison between lipegfilgrastim and pegfilgrastim in breast cancer patients and in accordance with the Cochrane Collaboration guidelines (The Cochrane Collaboration, 2011).

The aim of this study was to calculate the cost-utility of lipegfilgrastim as compared to pegfilgrastim in reducing the incidence of CIN and related complications in patients with breast cancer who received a four-cycle chemotherapy regimen of 21 days each. The perspective taken was that of the Belgian third-party payer (the National Institute for Health and Disability Insurance–INAMI/RIZIV) over a lifetime horizon. In contrast to current economic evaluations comparing G-CSF vs. non-G-CSFs or long-acting G-CSF (e.g., pegfilgrastim) vs. short-acting G-CSF (e.g., filgrastim) that solely track FN events during chemotherapy cycles (Lyman et al., 2009; Whyte et al., 2011; Fust et al., 2014, 2015; Kulikov et al., 2016), the approach taken in this study broadens the scope to additional CIN complications including incidence of SN, infection and chemotherapy dose delay. These are clinically meaningful outcomes considered in clinical guidelines, health technology appraisals and pharmacoeconomic studies (Aapro et al., 2006; Rutkowski et al., 2010; SMC. Scottish Medicines Consortium, 2011; Therapeutic Goods Administration, 2011; Johnson et al., 2014; Massoudi et al., 2017). In particular, SN has been shown to be significantly associated with both dose delay and relative dose intensity (RDI) less than 85% (Pettengell et al., 2008).

## Material and methods

### Study approach

A mathematical model was developed in Excel (Microsoft Corp., Redmond, WA) to estimate the lifetime costs and quality-adjusted life years (QALYs) for lipegfilgrastim and pegfilgrastim. Two Markov models, one tracking on-chemotherapy cycles and CIN-related complications (model 1) and one capturing the impact of RDI on long-term survival (model 2), compared cumulative costs and outcomes of lipegfilgrastim vs. pegfilgrastim based on two hypothetical cohorts of 1,000 women, aged 59 years old, 38.6, 47.5. and 13.9% having stage II, III, and IV breast cancer, respectively (proportions based on the relative distribution of patients with high risk stage II, stage III, and IV breast cancer as per the intent-to-treat population in Bondarenko et al., 2013). The models incorporated three sets of data. Clinical data, such as health state transition probabilities, were obtained from Bondarenko et al. and other published studies (Bodey et al., 1966; Leonard et al., 2003; Kuderer et al., 2006; Chirivella et al., 2009; Freifeld et al., 2011; Whyte et al., 2011; Fust et al., 2014; Wang et al., 2015). Transition probabilities and resource use data pertaining to treatment guidelines and clinical practice in a Belgian health care setting were validated or adjusted by Belgian clinicians in a modified Delphi survey. Unit cost data were derived from publicly accessible governmental databases (CBIP-BCFI BCfPI, 2015; National Institute for Health and Disability Insurance (RIZIV-INAMI), 2015b).

### Model structure

**Figure 1:**
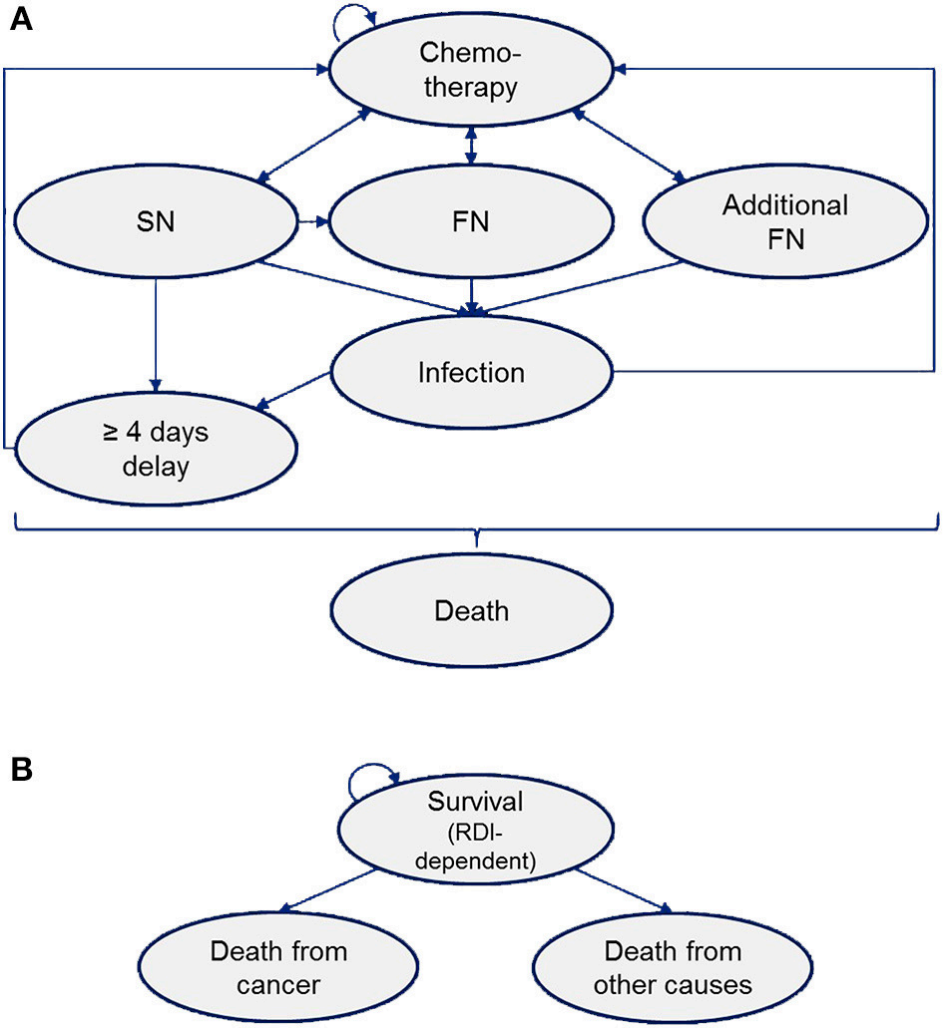
Model structure. **(A)** Model 1: Chemotherapy Markov model. **(B)** Model 2: Post-chemotherapy Markov model. The cycle length of the models were 3 weeks and 1 year for the on-chemotherapy model (model 1) and post-chemotherapy model (model 2), respectively. SN, severe neutropenia; FN, febrile neutropenia; RDI, relative dose intensity.

The model structure is presented in Figure 1. All patients enter the model in the state labeled “chemotherapy” upon administration of chemotherapy agents, such as docetaxel and anthracycline, and G-CSFs, pegfilgrastim or lipegfilgrastim on day 2 of each chemotherapy cycle. Dependent on risk factors, patients either move to chemotherapy-related complication health states, based on transition probabilities, or remain in their current health state. Patients with SN or infection were considered at risk for chemotherapy delay (Pettengell et al., 2012). Deaths associated with SN, FN and infection were considered in model 1. Post-chemotherapy deaths from breast cancer and other causes were considered in model 2, taking into consideration the impact of RDI (as a result of SN and FN events) on survival. Table 1 summarizes the model parameters.

**Table 1:** Summary of model parameters.

**Parameter**	**Base case value**	**Distribution**	**Source**
**Transition Probabilities**			
Baseline risk of FN associated with pegfilgrastim	0.032	Beta (alpha:3.000, beta: 91.000)	Bondarenko et al., 2013
OR of FN with lipegfilgrastim vs. pegfilgrastim	0.981	Log-normal (mean of logs: −0.020, SD of logs: 0.750)	Wang et al., 2015
RR of FN event in chemotherapy cycle ≥ 2 vs. cycle 1	0.213	Log-normal (mean of logs: −1.562, SD of logs: 0.164)	Whyte et al., 2011; Fust et al., 2014
**Base Risk of FN**^a^			
Cycle 1, lipegfilgrastim vs. pegfilgrastim	0.019 vs. 0.019	Based on previous inputs	
Cycle 2, lipegfilgrastim vs. pegfilgrastim	0.004 vs. 0.004	Based on previous inputs	
Cycle 3, lipegfilgrastim vs. pegfilgrastim	0.004 vs. 0.004	Based on previous inputs	
Cycle 4, lipegfilgrastim vs. pegfilgrastim	0.004 vs. 0.004	Based on previous inputs	
Risk of additional FN events in subsequent cycles	9.089	Log-normal (mean of logs: 2.188, *SD* of logs: 0.196)	Whyte et al., 2011; Fust et al., 2014
Risk of infection in patients with FN	0.300	Beta (alpha: 30.811, beta: 71.893)	Freifeld et al., 2011
Risk of death in patients with FN	0.036	Beta (alpha: 25.461, beta: 3,611.799)	Kuderer et al., 2006; Whyte et al., 2011
**Risk of SN**			
Cycle 1, lipegfilgrastim vs. pegfilgrastim	0.436 vs. 0.511	Beta (alpha: 41.000 vs. 48.000, beta: 53.000 vs. 46.000)	Bondarenko et al., 2013
Cycle 2, lipegfilgrastim vs. pegfilgrastim	0.085 vs. 0.215	Beta (alpha: 8.000 vs. 20.000, beta: 86.000 vs. 73.000)	Bondarenko et al., 2013
Cycle 3, lipegfilgrastim vs. pegfilgrastim	0.086 vs. 0.121	Beta (alpha: 8.000 vs. 11.000, beta: 85.000 vs. 80.000)	Bondarenko et al., 2013
Cycle 4, lipegfilgrastim vs. pegfilgrastim	0.122 vs. 0.121	Beta (alpha: 11.000 vs. 11.000, beta: 79.000 vs. 80.000)	Bondarenko et al., 2013
**Risk of FN if SN (Based on ANC)**^b^			
Cycle 1, lipegfilgrastim vs. pegfilgrastim	0.363 vs. 0.384	Gamma (alpha: 0.900 vs. 0.600, beta: 1.400 vs. 1.700)	Bodey et al., 1966; Bondarenko et al., 2013
Cycle 2, lipegfilgrastim vs. pegfilgrastim	0.305 vs. 0.313	Gamma (alpha: 1.500 vs. 1.600, beta: 1.700 vs. 1.300)	Bodey et al., 1966; Bondarenko et al., 2013
Cycle 3, lipegfilgrastim vs. pegfilgrastim	0.335 vs. 0.300	Gamma (alpha: 1.300 vs. 1.800, beta: 1.900 vs. 1.100)	Bodey et al., 1966; Bondarenko et al., 2013
Cycle 4, lipegfilgrastim vs. pegfilgrastim	0.271 vs. 0.311	Gamma (alpha: 2.500 vs. 1.800, beta: 1.100 vs. 1.300)	Bodey et al., 1966; Bondarenko et al., 2013
**Risk of Chemotherapy Delay if SN**^c^			
Cycle 2, lipegfilgrastim vs pegfilgrastim	0.238 vs. 0.216	Based on the Bayes' theorem	Pettengell et al., 2008; Bondarenko et al., 2013
Cycle 3, lipegfilgrastim vs. pegfilgrastim	0.256 vs. 0.0307	Based on the Bayes' theorem	Pettengell et al., 2008; Bondarenko et al., 2013
Cycle 4, lipegfilgrastim vs. pegfilgrastim	0.073 vs. 0.126	Based on the Bayes' theorem	Pettengell et al., 2008; Bondarenko et al., 2013
**Risk of Infection in Patients with SN**^b^			
Cycle 1, lipegfilgrastim vs. pegfilgrastim	0.363 vs. 0.384	Gamma (alpha: 0.900 vs. 0.600, beta: 1.400 vs. 1.700)	Bodey et al., 1966; Bondarenko et al., 2013
Cycle 2, lipegfilgrastim vs. pegfilgrastim	0.305 vs. 0.313	Gamma (alpha: 1.500 vs. 1.600, beta: 1.700 vs. 1.300)	Bodey et al., 1966; Bondarenko et al., 2013
Cycle 3, lipegfilgrastim vs. pegfilgrastim	0.335 vs. 0.300	Gamma (alpha: 1.300 vs. 1.800, beta: 1.900 vs. 1.100)	Bodey et al., 1966; Bondarenko et al., 2013
Cycle 4, lipegfilgrastim vs. pegfilgrastim	0.271 vs. 0.311	Gamma (alpha: 2.500 vs. 1.800, beta: 1.100 vs. 1.300)	Bodey et al., 1966; Bondarenko et al., 2013
**Risk of Chemotherapy Delay if Infection**^d^			
Cycle 2, lipegfilgrastim vs. pegfilgrastim	0.291 vs. 0.236	Based on the Bayes' theorem	Pettengell et al., 2008; Bondarenko et al., 2013
Cycle 3, lipegfilgrastim vs. pegfilgrastim	0.314 vs. 0.335	Based on the Bayes' theorem	Pettengell et al., 2008; Bondarenko et al., 2013
Cycle 4, lipegfilgrastim vs. pegfilgrastim	0.090 vs. 0.138	Based on the Bayes' theorem	Pettengell et al., 2008; Bondarenko et al., 2013
Risk of death if chemotherapy delay	0.001		Chirivella et al., 2009
Risk of death in patients with SN	0.0005	Beta (alpha: 14.196, beta: 190.064)	Delphi panel
Risk of death if infection	0.036	Beta (alpha: 42.808, beta: 1146.315)	Delphi panel
Risk of hospitalization if FN	0.800	Beta (alpha: 0.364, beta: 0.091)	Delphi panel
Proportion of patients with FN managed in outpatient setting	1–risk of hospitalization if FN		
Proportion of patients with SN managed in outpatient setting^e^	1		Assumption
Risk of hospitalization if infection	1		Delphi panel
Risk of RDI < 85% if FN	0.420	Beta (alpha: 9.784, beta: 13.511)	(Pettengell et al., 2008)
RR RDI < 85% for age ≥ 65 vs. < 65 years old	1.380	Log-normal (mean of logs: 0.322, SD of logs: 0.027)	Shayne et al., 2006; Lyman, 2009
Risk of RDI < 85%, age < 65 years old, no FN	0.247	Beta (alpha: 18.129, beta: 55.267)	Shayne et al., 2006; Whyte et al., 2011
OR of RDI < 85%, history of FN vs. no history of FN	1.580	95% CI: 1.200 – 2.100	Shayne et al., 2006
Risk of RDI < 85%, FN naïve, age < 65 years old, lipegfilgrastim vs. pegfilgrastim	0.044 vs. 0.044	Calculated based on aforementioned RDI related values	
Risk of RDI < 85%, FN history, age < 65 years old, lipegfilgrastim vs. pegfilgrastim	0.057 vs. 0.057	Calculated based on aforementioned RDI related values	
Risk of RDI < 85%, FN naïve, age ≥ 65 years old, lipegfilgrastim vs. pegfilgrastim	0.060 vs. 0.060	Calculated based on aforementioned RDI related values	
Risk of RDI < 85%, FN history, age ≥ 65 years old, lipegfilgrastim vs. pegfilgrastim	0.079 vs. 0.079	Calculated based on aforementioned RDI related values	
Risk of RDI < 85% if SN	0.280	Beta (alpha: 40.020, beta: 102.909)	(Pettengell et al., 2008)
Risk of RDI < 85%, SN, age < 65 years old, lipegfilgrastim vs. pegfilgrastim	0.118 vs. 0.118	Calculated based on aforementioned RDI related values	
Risk of RDI < 85%, SN, age ≥ 65 years old, lipegfilgrastim vs. pegfilgrastim	0.162 vs. 0.162	Calculated based on aforementioned RDI related values	
**Utility Values**			
Chemotherapy/G-CSF	0.700	Beta (alpha: 12.633, beta: 5.414)	(Ramsey et al., 2009)
SN	0.420	Beta (alpha: 17.769, beta: 24.538)	Gold et al., 2009
Disutility, treatment delay	0.500	Beta (alpha: 1.500, beta: 1.500)	Chan et al., 2012
FN inpatient	0.330	Beta (alpha: 35.701, beta: 72.484)	Ramsey et al., 2009
FN outpatient^f^	0.380	Beta (alpha: 27.176, beta: 44.339)	Assumption
Infection	0.330	Same as FN inpatient	Assumption
Breast cancer in years 1–5	0.860	Beta (alpha:3.742, beta: 0.609)	Ramsey et al., 2009
Breast cancer in years > 5	0.960	Beta (alpha: 1.399, beta: 0.058)	Ramsey et al., 2009
**Costs**			
Chemotherapy lipegfilgrastim vs. pegfilgrastim^g^	2,098 vs. 2,098	Gamma (alpha: 44.444 vs. 44.444, beta: 47.211 vs. 47.211)	http://www.bcfi.be/
FN (Inpatient Cost)^h^			
Ward cost	473	Gamma (alpha: 17.472, beta: 27.045)	INAMI/RIZIV, Delphi panel/
Length of stay	6	Gamma (alpha: 7.111, beta: 0.844)	Gladkov et al., 2016
Lipegfilgrastim vs. pegfilgrastim^i^	2,599 vs. 2,599	Calculate by multiplying the length of stay by the ward cost.	Conservative assumption for lipegfilgrastim
**Infection if FN, per day**^j^^,^^k^			
Cycle 1, lipegfilgrastim vs. pegfilgrastim	3,284 vs. 3,284	Gamma (alpha: 44.444 vs. 44.444, beta: 73.894 vs. 73.894)	BCFI, INAMI/RIZIV, Delphi panel
Cycle 2, lipegfilgrastim vs. pegfilgrastim	3,282 vs. 3,282	Gamma (alpha: 44.444 vs. 44.444, beta: 73.894 vs. 73.894)	BCFI, INAMI/RIZIV, Delphi panel
Cycle 3, lipegfilgrastim vs. pegfilgrastim	3,282 vs. 3,282	Gamma (alpha: 44.444 vs. 44.444, beta: 73.894 vs. 73.894)	BCFI, INAMI/RIZIV, Delphi panel
Cycle 4, lipegfilgrastim vs. pegfilgrastim	3,282 vs. 3,282	Gamma (alpha: 44.444 vs. 44.444, beta: 73.894 vs. 73.894)	BCFI, INAMI/RIZIV, Delphi panel
**Infection if SN, Per Day**^k^			
Cycle 1, lipegfilgrastim vs. pegfilgrastim	3,380 vs. 3,380	Gamma (alpha: 44.444 vs. 44.444, beta: 76.041 vs. 76.041)	BCFI, INAMI/RIZIV, Delphi panel
Cycle 2, lipegfilgrastim vs. pegfilgrastim	3,372 vs. 3,372	Gamma (alpha: 44.444 vs. 44.444, beta: 76.041 vs. 76.041)	BCFI, INAMI/RIZIV, Delphi panel
Cycle 3, lipegfilgrastim vs. pegfilgrastim	3,372 vs. 3,372	Gamma (alpha: 44.444 vs. 44.444, beta: 76.041 vs. 76.041)	BCFI, INAMI/RIZIV, Delphi panel
Cycle 4, lipegfilgrastim vs. pegfilgrastim	3,372 vs. 3,372	Gamma (alpha: 44.444 vs. 44.444, beta: 76.041 vs. 76.041)	BCFI, INAMI/RIZIV, Delphi panel
Outpatient setting, FN	44	Beta (alpha 16,056; beta 2.724)	BCFI, INAMI/RIZIV, Delphi panel

a *Estimates dependent on the risk estimate used (i.e., RR of 0.981 as per Wang et al., 2015 or OR of 0.340 as per Bond et al., 2015). Calculation are herein based on Wang et al*.

b *A gamma distribution was assigned to the ANC based on the mean ANC published by Bondarenko et al. for lipegfilgrastim and pegfilgrastim and 1,000 iterations were generated. Then, ANC < 0.5 × 109/L were used to derived the risk of FN/infection, thanks to the correlation established by Bodey et al. between granulocyte levels and risk of infection. It was then conservatively assumed that the risk of FN is the same as the risk of infection in patients with SN. Patients with SN without fever, signs, or symptoms ofinfection do not receive treatment in the hospital, but are followed up for potential development of fever at home*.

c The risk of delay was calculated based on the Bayes' theorem (conditional probability): $\frac{\text{P}\left(\text{SN}|\text{delay}\right)}{\text{P}\left(\text{Non}-\text{SN}|\text{delay}\right)}=\frac{\text{P}\left(\text{delay}|\text{SN}\right)}{\text{P}\left(\text{delay}|\text{Non}-\text{SN}\right)}*\frac{\text{P}\left(\text{SN}\right)}{\text{P}\left(\text{Non}-\text{SN}\right)}$; P(Non−SN|delay) = 1−P(SN|delay) and P(Non−SN) = 1−P(SN). The probability of delay in patients with SN and the probability of delay in patients without SN were as follow: P(delay|SN) = 0.42 and P(delay|Non−SN) = 0.32 (Pettengell et al., 2008). The probability of SN across all cycles was 0.500 and 0.585 for lipegfilgrastim and pegfilgrastim, respectively (Bondarenko et al., 2013).

d *The same approach as the risk of delay in patients with FN was considered. However, it was assumed that the risk of delay in patients with infection is the same as the risk of delay in patients with FN. The probability of delay in patients with FN and the probability of delay in patients without FN were as follow: P(delay|FN) = 0.42 and P(delay|Non−FN) = 0.32 (Pettengell et al., 2008). The probability of FN across all cycles was 0.635 and 0.649 for lipegfilgrastim and pegfilgrastim, respectively. These probabilities were calculated from the baseline risk of FN (therefore dependent on risk estimate used as per Wang et al. or Bond et al. – here Wang et al.), the risk of additional FN in subsequent cycles and the estimated risk of FN in patients with SN*.

e *Patients with SN without fever, signs, or symptoms ofinfection do not receive treatment in the hospital, but are followed up for potential development of fever at home. No cost was imputed for the management of SN in the outpatient setting*.

f *The difference (i.e., 0.05) between utilities for patients hospitalized for FN vs. those treated in an outpatient setting as per Lathia et al. (2013) was simply applied*.

g *Treatment costs were composed of the unit cost of G-CSF (lipegfilgrastim or pegfilgrastim) : €1,169; cost of chemotherapy agents docetaxel (20 mg/ml): €68; and doxorubicine TEVA (10 mg/5 ml): €8 (CBIP-BCFI BCfPI, 2015); and G-CSF administration cost: €8 (Annemans et al., 2001). G-CSF administration costs were inflated to 2015 using the Harmonized Index of Consumer Price for Health (Federal Reserve Bank of St.Louis, 2015). Chemotherapy agent related costs were adjusted to a female patient with a BMI of 26 kg/m^2^ and a weight of 73.9 kg*.

h *Of note, in the model, the costs of empirical antibiotic treatment was also considered. If a patient is admitted at the hospital with FN, “empiric” antibiotic therapy is initiated at admission. Piperacilline-tazobactam 4 × 4 g/d is commonly used and treatment is stopped when until restitution of neutrophils > 1,000/mm^3^ AND free of fever after 48 h AND negative bacterial cultures. Median time of treatment duration with piperacilline-tazobactam for breast cancer patients is 4 days. The ward cost was calculated as the average of the daily cost for all Belgian hospitals*.

i *The time in hospital, day, mean (SD) was 1 (0) and 5.5 (0.7) for lipegfilgrastim and pegfilgrastim based on the Bondarenko head-to-head trial, respectively (Gladkov et al., 2016). This would have resulted in FN hospital costs of 473 vs. 2,599 for lipegfilgrastim vs. pegfilgrastim. Because the only patient who was hospitalized for FN was excluded for protocol violation (Bondarenko et al., 2013), we conservatively assumed that the length of stay for lipegfilgrastim would be the same as of pegfilgrastim. The value of 5.5 days was rounded up to 6 days*.

j *The duration of antibiotic use was considered to be 5 days for both treatment arms given that no significant difference in antibiotic use have been demonstrated between the two G-CSFs (Bondarenko et al., 2013; Mhaskar et al., 2014; Gladkov et al., 2016)*.

k *Weighted average calculated based on the following prevalence of infection: bacteremia: 60.0%, sepsis: 22.5%, pneumonia: 8.1% and fungal infection: 9.4% (assumption)*.

*BCFI, Belgian Centre for Pharmacotherapeutic Information; FN, febrile neutropenia; OR, odds ratio; RR, relative risk; SN, severe neutropenia. The transition probabilities were validated in a modified Delphi panel and are reflective of current clinical practice and management of chemotherapy-induced neutropenia and related complications in patients with breast cancer in Belgium*.

### Clinical data and utilities

#### On-chemotherapy model (model 1)

The model incorporates the incidence of SN in patients receiving lipegfilgrastm as compared to pegfilgrastim in each cycle of chemotherapy, with a significantly lower incidence of SN for lipegfilgrastim in cycle 2 (8.5 vs. 21.5%; *p*-value = 0.013; Bondarenko et al., 2013). The risk of FN was estimated for patients with SN transitioning to FN and for those at a risk of FN subsequent to chemotherapy (base FN risk). The risk of FN in case of SN was estimated based on the relationship between granulocytes level and infection (Bodey et al., 1966), and the probability of ANC less than 0.5 × 10^10^/L (Bondarenko et al., 2013). It was then conservatively assumed that the risk of FN is the same as the risk of infection.

The base risk of FN for lipegfilgrastim was calculated from the risk of FN in the pegfilgrastim group to which was applied a risk estimate for FN for lipegfilgrastim vs. pegfilgrastim. This adjustment was considered appropriate given that in the Bondarenko study, the only patient who had FN in the lipegfilgrastim arm was excluded for protocol violation (Bondarenko et al., 2013). As mentioned earlier, two meta-analyses reported two different risk estimates for FN for lipegfilgrastim vs. pegfilgrastim. Wang et al. reported an OR of 0.98 (95% CI: 0.21–4.53) based on a direct comparison between lipegfilgrastim and pegfilgrastim in breast cancer patients. The indirect comparison included trials vs. placebo or no-G-CSF in different patient populations (breast cancer, colorectal cancer, non-Hodgkin lymphoma, lung and ovarian cancer), resulting in an OR of 2.00 (95% CI: 0.47–8.11). The result of the combination of direct and indirect comparisons was between the results of the direct and the indirect comparison, with an OR of 1.39 (0.54–3.50) (Wang et al., 2015). Bond et al., on the other hand, reported a RR of 0.34 (95% CI: 0.05–2.14) in a direct comparison between lipegfilgrastim vs. pegfilgrastim (Bond et al., 2015). Given (i) the recommendations of the Cochrane Handbook for Systematic Reviews of Interventions stating that direct comparison should take precedence over indirect comparison in forming conclusions (The Cochrane Collaboration, 2011), (ii) the application of inconsistent indirect or mixed methods by Wang et al. that led to potentially biased risk estimates not in the alignment with the head to head trial results (Lehmacher et al., 2016), risk estimates from direct comparison in breast cancer patients were considered relevant in the current study. The base analyses were performed using an OR of 0.98 (95% CI: 0.21–4.53) and a RR of 0.34 (95% CI: 0.05–2.14). All other transition probabilities were derived from literature or adjusted for lipegfilgrastim and pegfilgrastim wherever needed.

#### Post-chemotherapy model (model 2)

RDI is the ratio of the actual dose of chemotherapy delivered to the intended dose of the standard chemotherapy regimen over a specific time. Decreases in RDI can be results of dose delays and dose reductions. Although controversial, the relationship between RDI and patient outcomes is well described in the literature (Bonadonna et al., 2005; Pettengell et al., 2008; Bretzel et al., 2009; Chirivella et al., 2009; Aapro et al., 2011); and several studies address the significance of achieving an RDI of more than 85% (Bonadonna et al., 2005; Lyman, 2008). Age, SN and FN are predictors of receiving RDI less than 85% (Shayne et al., 2006; Pettengell et al., 2008; Whyte et al., 2011). Gladkov et al. reported that at least 98% of patients in the lipegfilgrastim and the pegfilgrastim received the planned chemotherapy dose (Gladkov et al., 2016). Consequently, the risk of RDI less than 85% was assumed to be the same for both G-CSFs (Pettengell et al., 2008). It was further adjusted for age, incidence of SN, incidence of FN and FN history (Leonard et al., 2003; Shayne et al., 2006; Chirivella et al., 2009; Lyman et al., 2009; Whyte et al., 2011).

The impact of RDI on survival was considered next. The estimation of the risk of death if RDI less than 85% was as *per* Whyte et al.'s formula that is based on the risk of RDI less than 85% when experiencing neutropenic events and the survival hazard ratio (Whyte et al., 2011). The literature suggests that for anthracycline-based regimens, 32% of patients who experienced neutropenic events receive RDI less than 85%, compared with 7% for those who do not experience these events (Leonard et al., 2003). Additionally, a hazard ratio of 1.32 was seen for survival associated with an RDI ≥ 85% vs. an RDI < 85% (Lyman et al., 2009). The model applies this hazard ratio to the survival of patients with low RDI in the first 5 years of the post-chemotherapy model. After and for the remainder of their lifetime, all-cause mortality as *per* the general population applied as it was assumed that breast cancer survivors were cured after 5 years. Breast cancer specific mortality data by stage and age were obtained from the Belgian Cancer Registry and all-cause mortality data from Belgian life tables (data on file).

### Utility values

Health utilities, which vary between 0 (death) and 1 (ideal health) were used to calculate the QALYs. Utility values were obtained from the literature for breast cancer during chemotherapy, SN, FN/infection, breast cancer survivor during years one to five and breast cancer survivor after year five (Table 1). A disutility factor was assigned to treatment delay. QALYs were calculated by multiplying life years gained over the course of the chemotherapy and post-chemotherapy models by utility values.

### Resource use and cost data

#### Delphi panel survey

Resource use data were obtained from a random selection of 64 oncologists and hematologists through a modified Delphi survey methodology. The Delphi survey was conducted in two consecutive rounds. Based on identified treatment pathways, a questionnaire was developed with closed-ended questions about medication, laboratory tests, and diagnostic procedures and open-ended questions about additional information (e.g., to obtain empirical data as opposed to protocol-driven clinical research data). In the first round, the survey was conducted via a web-based application, SurveyMonkey®, yielding 11 responses out of 64 experts recruited. The results were analyzed as median, 25th, and 75th percentile outcomes. In the second round, results obtained were presented to the 11 participating respondents via email or by means of a face-to-face interview to validate/adjust the results, yielding 7 responses with narrower variances between outcomes.

#### Costs associated with severe neutropenia, febrile neutropenia, infection and delay

Resource use related to: G-CSFs (lipegfilgrastim and pegfilgrastim), medication (antibiotics and antifungals), chemotherapy regimen, laboratory tests, diagnostic procedures (e.g., X-ray, CT-scan), type and duration of hospitalization (e.g., emergency room, oncology ward) were taken into account. Medical procedures related costs were obtained from the Nomensoft database on the INAMI/RIZIV website (National Institute for Health and Disability Insurance (RIZIV-INAMI), 2015a). Drug cost data (in 2015 euros – €), which were derived from the Belgian Center of Pharmacotherapeutic Information website (CBIP-BCFI BCfPI, 2015), were collected for the drugs currently prescribed for the treatment of SN, FN and associated complications. The modified Delphi survey yielded data about the use of antibiotics and antifungals for the four most common infections following SN and FN, being bacteremia, sepsis, pneumonia, and fungal infections (Kuderer et al., 2006). An average antibiotics treatment duration of 5 days for hospitalized patients with FN was considered in both treatment arms (Mhaskar et al., 2014). Treatment costs for antibiotics and anti-fungal medication were computed as a weighted average of the cost per treatment course, taking relative market share into consideration.

The per diem cost of hospitalization was computed from data obtained from the NIHDI website (National Institute for Health and Disability Insurance (RIZIV-INAMI), 2015b) taking the mean of all listed acute care facility admission costs in Belgium. Daily costs for hospital stay, laboratory and microbiological tests as well as radiology procedures were calculated and adjusted to length of stay.

With respect to FN, inpatient and outpatient costs were considered. The hospitalization length of stay was derived from Gladkov et al. (2016), whilst it was assumed that the risk of hospitalization was similar between the two G-CSFs and did not differ between cycles. With the FN outpatient costs, it was assumed that patients were treated for seven to 10 days with per oral amoxicillin/clavulanic acid 2 × 2 g/day as per the clinical practice (data on file).

Finally, there were no costs imputed for the management of SN (only in the outpatient setting) and of chemotherapy delay. There were also no costs associated with the post-chemotherapy model.

### Base case analysis

Model parameters base values were defined and differences on costs and QALYs between the lipegfilgrastim group and the pegfilgrastim group calculated. The incremental cost-effectiveness ratio (ICER), calculated as the ratio of the incremental costs to the incremental QALYs between lipegfilgrastim and pegfilgrastim, was calculated when appropriate. Analyses were performed over a lifetime horizon. Costs and QALYs were discounted at a rate of 3 and 1.5%, respectively following guidelines of the Belgian Health Care Knowledge Centre (KCE) (Cleemput et al., 2012). Effectiveness was also reported in terms of life-years gained (LYG) that were not discounted since they were expected to be valuable in the future as at present. In comparison to LYG, QALYs involve both quantitative and qualitative components that are likely to be less valuable in the future as at present, and are also likely to be subject to higher uncertainty (Peura et al., 2008).

### One-way sensitivity analysis

One-way sensitivity analysis (OWSA) and probabilistic sensitivity analyses (PSA) were conducted to test both parameter and model uncertainty. In the OWSA, sensitivity ranges of parameters included either 95% CIs, where data was available, or the estimated variability of ±30% of the mean value. Tornado diagrams were used to present the results of the OWSA and to depict the parameters that mostly affect both costs and QALYs. With the PSA, the impact of joint uncertainty among all input parameters on cost-effectiveness outcomes was captured by assigning a probability distribution to each parameter and using 5,000 random samples from parameter distributions. A cost-effectiveness acceptability curve (CEAC) was subsequently drawn, showing the probability of lipegfilgrastim being cost-effective for various levels of willingness to pay (WTP) per QALY gained.

### Price threshold analysis

A price threshold analysis was subsequently conducted to determine the upper limit of the price of lipegfilgrastim to be considered cost-effective, using a WTP of €30,000 per QALY gained (corresponding approximately to the Belgian Gross Domestic Product). The analysis was carried out across breast cancer stages and age bands of 35–45, 45–55, 55–65, 65–75, and over 75 years. The analysis was based on the net monetary benefit, which is the difference between monetized benefits and costs between lipegfilgrastim and pegfilgrastim. Interventions with a positive net monetary benefit are deemed cost-effective as the associated cost is less than the value of the additional benefit achieved.

## Results

### Base case analysis

The average costs and QALYs of primary prophylaxis with lipegfilgrastim compared to pegfilgrastim for patients with stages II-IV breast cancer are presented in Table 2 Cost-effectiveness results. Results are presented for an OR of 0.98 and a RR of 0.34 for the risk of FN for lipegfilgastim compared to pegfilgrastim. With an OR of 0.98, the average costs per patient and QALYs for lipegfilgrastim were €9,845 and 13.977 compared to €10,208 and 13.925 for pegfilgrastim. Life expectancy in the lipegfilgrastim group was further increased by 0.058 years on average (i.e., 21 life-days gained) compared with pegfilgrastim. Similarly, when using a RR of 0.34, lipegfilgrastim was the dominant strategy with lower costs and higher QALYs compared to pegfilgrastim. In the analysis of LYG, an average increase by 0.059 years (i.e., 22 life-days gained) was estimated in the lipegfilgrastim group compared to the pegfilgrastim group. Overall, these results indicated that lipegfilgrastim was a dominant strategy in the base case. Consequently, the ICER was not calculated. Given the consistency in conclusions when using either an OR of 0.98 or a RR of 0.34 for the risk of FN for lipegfilgrastim compared to pegfilgrastim, following results will be limited to the analyses conducted with an OR of 0.98.

**Table 2:** Cost-effectiveness results for lipegfilgrastim compared to pegfilgrastim.

	**Results based on Wang et al.'s risk estimate for FN, lipeg vs. peg: OR** = **0.98)**			**Results based on Bond et al.'s risk estimate for FN, lipeg vs. peg: RR** = **0.34)**		
	**Costs (€)**	**QALYs**	**LY**	**Costs (€)**	**QALYs**	**LY**
Lipegfilgrastim	9,845	13.977	21.204	9,796	13.997	21.210
Pegfilgrastim	10,208	13.925	21.145	10,205	13.944	21.151
Difference	−363	0.052	0.058	−410	0.054	0.059

*Analyses were performed with an odd ratio of 0.98 (direct comparison in patients with breast cancer as per Wang et al., 2015) and with a relative risk of 0.34 (direct comparison in patients with breast cancer as per Bond et al., 2015). Mean costs, QALYs and LY were the average of 5,000 simulated estimates. Numbers are rounded so some differences may appear to be off. LY, life years; OR, odds ratio; QALYs, quality-adjusted life years gained; RR, relative risk*.

In deterministic analyses, 41% of the cost-savings came from avoided infection cases and associated costs when comparing lipegfilgrastim with pegfilgrastim. Twenty eight percent of the cost-savings were associated with avoided SN cases, 21% with avoided FN cases and 10% with avoided chemotherapy delay. Conclusively, there were fewer patients who experienced CIN-related complications and associated costs in the lipegfilgrastim group compared to the pegfilgrastim group. The chemotherapy/G-CSF costs were offset by higher costs associated with CIN-related complications; and particularly with infection. The difference in QALYs was attributable to the difference in the number of patients in the chemotherapy/ G-CSF Markov state followed by infection, FN, SN and chemotherapy delay.

### One-way sensitivity analysis

**Figure 2:**
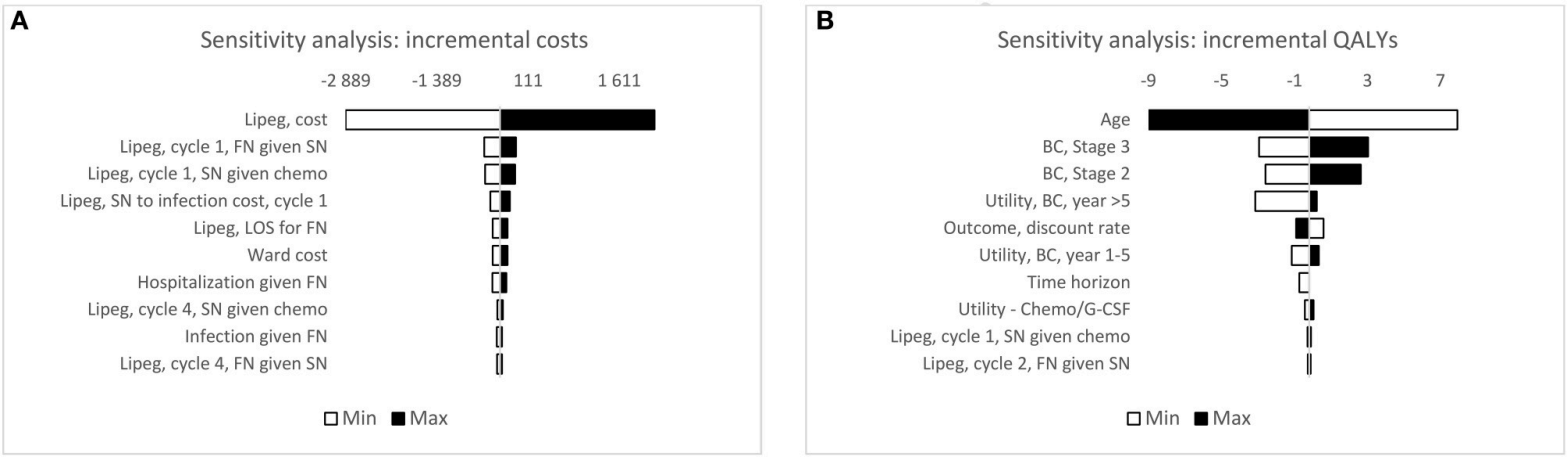
One-way sensitivity analysis for incremental cost and QALY. Tornado diagram displaying the results of the one-way sensitivity analysis. The diagram shows the correlation between ±30% variation in model parameters and the distribution of costs and QALYs. Only the top 10 influential parameters of the incremental costs and QALYs are displayed. BC, breast cancer; chemo, chemotherapy; FN, febrile neutropenia; G-CSF, granulocyte colony-stimulating factor; lipeg, lipegfilgrastim; QALYs, quality-adjusted life years; SN, severe neutropenia.

The impact of each of the model parameter on incremental costs and QALYs was explored in OWSA (Figure 2). For all scenarios within the possible ranges of model inputs, lipegfilgrastim remained less costly compared to pegfilgrastim; except when increasing the cost of lipegfilgrastim by 30%. The cost of lipegfilgrastim had the largest impact on the incremental costs, followed by the risk of FN in patients with SN in cycle 1 for lipegfilgrastim and the risk of SN following chemotherapy in cycle 1 for lipegfilgrastim. Incremental QALYs were most sensitive to variance in age, in the proportion of patients with stage III and II breast cancer and in the utility value for cancer survivors after year five. Overall, QALYs gained decreased as age increased; and increased as the proportion of patients with stage III and II breast cancer increased. Additionally, QALYs gained increased as the utility for cancer survivors after year five increased.

**Figure 3:**
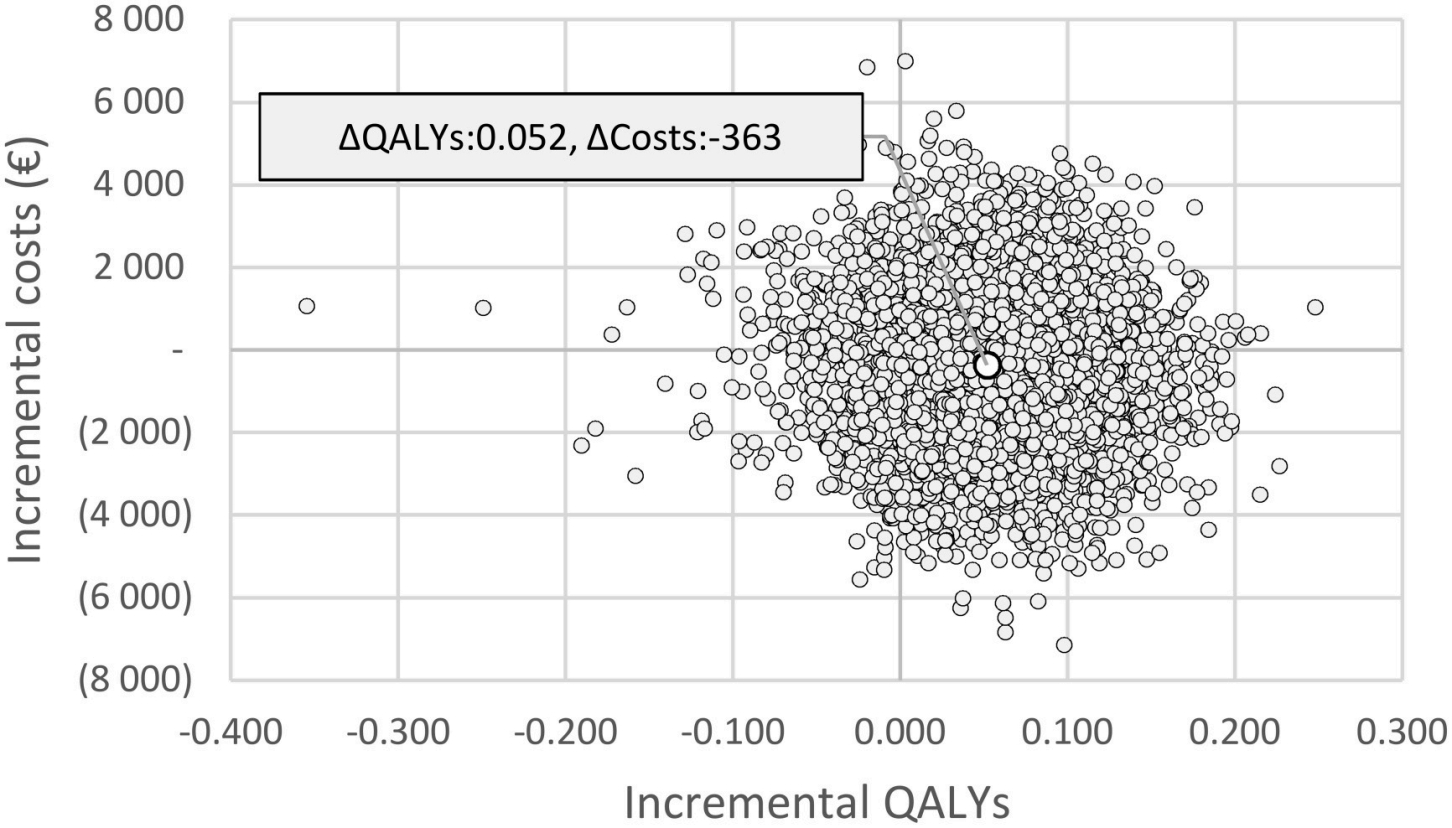
Cost-effectiveness plane for lipegfilgrastim compared to pegfilgrastim. Plot of the results of the probabilistic sensitivity analysis for 5,000 simulations. The x-axis represents the difference in QALYs and the Y-axis the difference in costs between lipegfilgrastim and pegfilgrastim. Seven percent of the estimates lie in the north-west quadrant (pegfilgrastim dominates), 36% in the north-east quadrant (lipegfilgrastim is more effective but more expensive), 7% in the south-west quadrant (lipegfilgrastim is cheaper but less effective) and 51% in the south-east quadrant (lipegfilgrastim dominates pegfilgrastim). Simulations spanned all four quadrants of the cost-effectiveness plane, indicating some level of uncertainty around the base case estimate (cost-savings of €363 and QALYs gained of 0.052). ΔQALYs, incremental quality-adjusted life years; ΔCosts, incremental costs.

**Figure 4:**
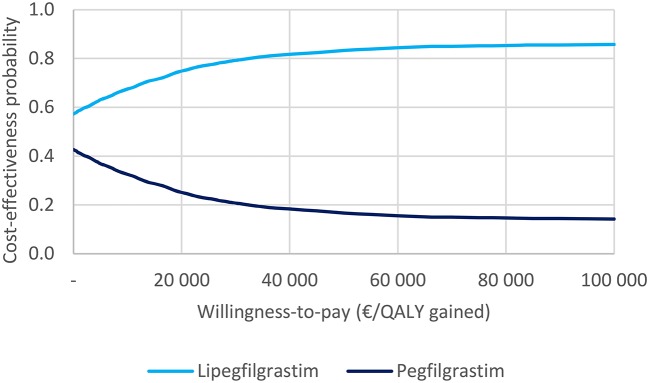
Cost-effectiveness acceptability curve.

The effect of joint parameter uncertainty on outcomes is illustrated in the cost-effectiveness plane. Figure 3 shows that lipegfilgrastim was associated with greater QALYs in 87% of the 5,000 simulations (northeast and southeast quadrants), and in over half of the simulations, lipegfilgrastim was dominant (southeast quadrant). Figure 4 indicates that the likelihood that lipegfilgrastim is cost-effective vs. pegfilgrastim increases as the WTP increases (i.e. gains in QALYs become increasingly rewarded), reaching 68, 79, and 83% at the WTP thresholds of €10,000, €30,000, and €50,000 per QALY gained, respectively. The probability that lipegfilgrastim is cost-saving is indicated by the probability at a WTP of zero (where no value is placed on the health benefits), which was 57%.

### Price threshold analysis

**Figure 5:**
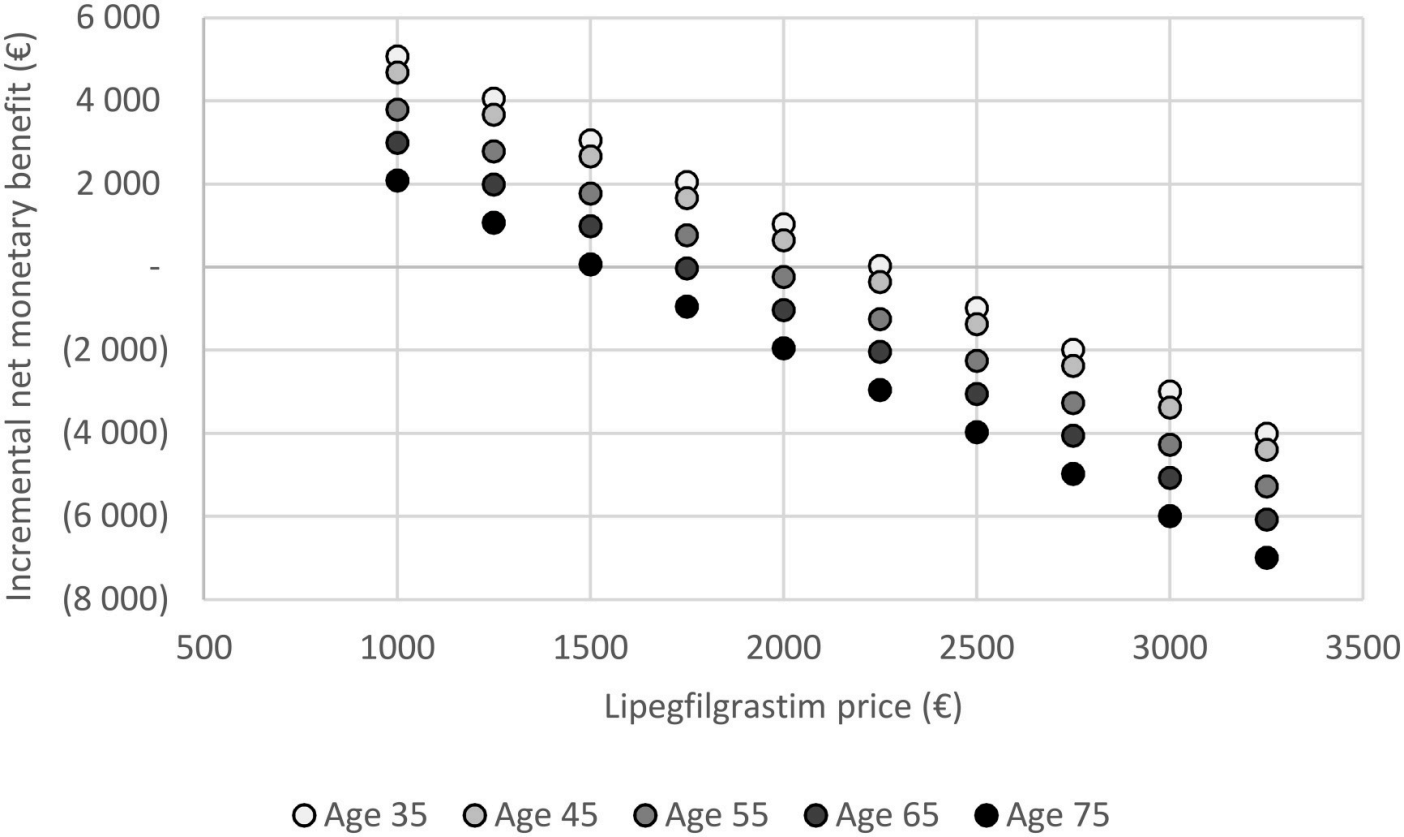
Price threshold analysis. Lipegfilgrastim price was varied across breast cancer stages and age bands of 35–45, 45–55, 55–65, and over 75 years old to examine efficient reimbursement prices. The analysis was conducted at a willingness to pay threshold of €30,000 per QALY gained. Each dot represents an incremental net monetary benefit value for a specific age at a given price point. Of note, the analysis was conducted keeping the following distribution of breast cancer stage constant: stage II: 38.6%, stage III: 47.5% and stage IV: 13.9%. Positive incremental net monetary benefit values indicate that lipegfilgrastim is cost-effective compared to pegfilgrastim. On the other hand, negative incremental net monetary benefit values indicate that lipegfilgrastim is not cost-effective compared to pegfilgrastim. Up to a price point of €1,500, all incremental net monetary benefit value are greater than zero. At higher price point, lipegfilgrastim shows to no longer be cost-effective for some age groups.

Figure 5 shows that at currently equivalent price with pegfilgrastim, lipegfilgrastim is dominant across all cancer stages and age bands. Lipegfilgrastim remained cost-effective across all cancer stages and age bands up to a price point of €1,500 at a WTP of €30,000 per QALY gained. Between €1,500 and €2,000, lipegfilgrastim is cost-effective in some age groups and is no longer cost-effective in all cancer stages and age bands at a price higher than €2,000.

## Discussion

Long-acting G-CSFs, lipegfilgrastim, and pegfilgrastim, are administered once per chemotherapy cycle to reduce the duration of SN and the incidence of FN. This study evaluated the cost-utility of lipegfilgrastim compared to pegfilgrastim, with a particular focus on the following outcome measures: incidence of SN, incidence of FN, infection, chemotherapy dose delay and RDI less than 85%. In addition, QALYs and LYs gained were captured as standard measures of effect.

This analysis has demonstrated that lipegfilgrastim is a more cost-effective strategy compared to pegfilgrastim over the lifetime of patients with stage II-IV breast cancer. The cost-savings came merely from the decrease in infection and SN cases. OWSA further indicated that cost-savings were sensitive to the risk of FN in patients with SN and the risk of SN following chemotherapy in cycle 1. Overall, these results align with the literature, which shows that SN places patients at high risk of infection and the risk of FN increases in direct proportion to the severity and duration of neutropenia and occurs most frequently early in a course of chemotherapy (Lyman et al., 2003; Lyman and Rolston, 2010; Freifeld et al., 2011; Saloustros et al., 2011). In our study, the risk of FN/infection in patients with SN was based on the relationship between granulocyte levels and the risk of infection as established by Bodey et al. in patients with acute leukemia (Bodey et al., 1966). Therefore, further studies on the relationship between granulocyte or ANC levels and the risk of FN/ infection in patients with breast cancer may be important. This could further be relevant when considering that lipegfilgrastim is associated with higher neutrophil count at the end of a chemotherapy cycle than pegfilgrastim (Bondarenko et al., 2013); suggesting that fewer patients might be at a risk of developing FN or to experience SN in subsequent chemotherapy cycles. Indeed, this was observed in the second chemotherapy cycle in Bondarenko et al.'s study. Finally, it should be mentioned that cost-savings were influenced the most by the variation in the cost of lipegfilgrastim. In Belgium, lipegfilgrastim and pegfilgrastim have the same price. The price threshold analysis suggested that lipegfilgrastim is cost-effective up to €1,500 across all age bands and cancer stages, compared to the current price of €1,169.

Effectiveness results were largely influenced by the patient age, breast cancer stage at diagnosis and the utility value for cancer survivors after year five. This was in line with previous cost-effectiveness studies reporting on these parameters as drivers of the cost-effectiveness of G-CSFs for the prevention of FN after chemotherapy (Lyman, 2009; Ramsey et al., 2009; Whyte et al., 2011). The impact of age on the cost-effectiveness results was expected given the age-dependent risk of RDI less than 85% (Shayne et al., 2006) and the relative impact on overall mortality. Further research on utility weights associated with breast cancer survivors after year five is required given the sensitivity of different cost-effectiveness studies on this parameter.

In a European survey relating to cancer therapy and neutropenic infections, Leonard reported that oncology nurses recognize that minimizing the risk of infection and FN as being important for achieving a successful outcomes in cancer therapy (Leonard, 2012). From a patient perspective, the majority reported having their chemotherapy delayed or changed as a result of neutropenia, infection or FN. Therefore, despite being broaden to major CIN-related complications, the value of our model derives merely from the most serious chemotherapy complications (i.e., SN, FN, and infection) health professionals deal with in their practice, those for which treatment algorithms and guidelines have been developed and that impact the most the patients (Aapro et al., 2006, 2011; Freifeld et al., 2011).

The results of our study aligned with Kulikov et al. (2016) but disagree with those obtained by Fust et al. (2015, 2016), who found pegfilgrastim to be dominant when compared to lipegfilgrastim. This difference in conclusion may arise from the study design. From a clinical standpoint, Fust et al.'s model was populated with an OR of 1.39 for the risk of FN in lipegfilgrastim vs. pegfilgrastim. As mentioned earlier, Wang et al. reported an OR of 1.39 as a result of an MTC and an OR of 0.98 as a result of the direct comparison between lipegfilgrastim and pegfilgrastim in patients with breast cancer only, both risk estimates being non-statistically significant (Wang et al., 2015). By not relying on the head-to-head trial comparing lipegfilgrastim and pegfilgrastim and by using an OR of 1.39 that stem from an indirect and MTC analyses that lack validity (Lehmacher et al., 2016), the incremental savings and utilities reported by Fust et al. may have been overestimated. Our analysis aligns with the requirements of leading institutions for health technology assessment (HTA) such as the Belgian KCE (Cleemput et al., 2012), the Canadian Agency for Drugs and Technology in Health (CADTH) (CADTH, 2006), the National Institute for Health and Clinical Excellence NICE, 2013) and the German Institute for Quality and Efficiency in Health Care (IQWIG) (IQWIG, 2012) that have a preference for data from head-to-head randomized controlled trials. Indirect treatment comparison methods may be used if data from head-to-head trials are not available.

Inconsistencies were further noted in Fust et al.'s model when considering costs. Consistent with the clinical effects and the potential for leucocytosis, white blood cell counts should be performed at regular intervals during therapy for both pegfilgrastim and lipegfilgrastim (Lonquex, 2015; Neulasta, 2015). However, this was applied only to lipegfilgrastim in Fust et al.'s model, at the rate of five complete blood counts vs. only one complete blood count prior chemotherapy with pegfilgrastim, potentially overestimating lipegfilgrastim treatment costs. Finally, outpatient FN costs in Fust et al. were as per the USA setting whilst our analysis reflects the Belgian clinical practice.

It could also be argued that the current model differs from Fust et al. in its structure and the selected population. Indeed, the current study focuses on stage II-IV breast cancer patients, whereas Fust et al. reported data on stage II breast cancer patients.

There are several limitations to the current study, the most important one being that currently, there is no direct clinical evidence that CIN leads to clinically recognized chemotherapy delay, lower RDI or poorer survival. However, it could be considered that this was well mitigated in the model as clinical data were used wherever possible. As such, similarity in the use of antibiotics or the fact that almost all patients had received the planned chemotherapy dose in both treatment arms were well reflected in the model. Next, resource use data were obtained *in fine* from only seven oncologist, holding the potential of a non-representative assessment of resource use. Post-chemotherapy costs were assumed to be zero given that the cost of G-CSFs and associated costs were captured in the chemotherapy model (model 1). In addition, there is limited data concerning the impact of RDI on resource utilization and associated costs. Furthermore, the treatment costs for bacteraemia, sepsis, pneumonia, and fungal infection were considered independently. However, such infections are not mutually exclusive. Other types of infection (urinary tract infections, skin and deep tissue infections, mucosal infections, upper respiratory tract infections, etc.) may occur. Sensitivity analyses indicated that costs for treating infections as a result of SN or FN events and the relative incidence of type of infection were not drivers of the incremental costs. Importantly, translating the granulocyte levels into a risk of infection was based on patients with leukemia (Bodey et al., 1966) given the lack of data in patients with breast cancer.

Our analysis considered the payer's perspective, whereas a societal perspective may be important in other circumstances (Weinstein et al., 1996). Including indirect costs, such as patient time, caregiver costs and lost productivity may improve the cost-effectiveness of lipegfilgrastim compared to pegfilgrastim. Finally, although patients with chemotherapy dose delay have about one percent risk of relapse (data on file), relapse was not captured in the present model.

In conclusion, this analysis shows that, for equivalent drug costs, lipegfilgrastim is a dominant strategy compared to pegfilgrastim for the management of patients with stage II-IV breast cancer. Lipegfilgrastim shows to be cost-effective up to €1,500 across all age bands and cancer stages, compared to the current price of €1,169.

## Author contributions

This study was carried out in collaboration with all authors. EA and EM conceived and designed the study in consultation with all authors. EA, EM, IJ, and SS were involved in the data collection. EA developed the model and carried out the data analysis. EA, EM, IJ and SS interpreted the results. EA, EM, and IJ wrote a full report based on which SS wrote the draft manuscript. This draft manuscript was revised by EA, EM, and IJ, and all authors approved the final version.

### Conflict of interest statement

EA, EM, and IJ are employees of Deloitte and SS is an employee of KU Leuven. Deloitte in collaboration with KU Leuven received an unconditional grant from TEVA to carry out this study.
